# Catarrh of the Bowels and Heat Stroke

**Published:** 1901-08

**Authors:** J. M. Gibb

**Affiliations:** Jamaica


### CATARRH OF THE BOWELS AND HEAT STROKE.

By J. M. Gibb, V.S.,
JAMAICA.

A red plough steer, eight years old, showed symptoms during work, staggered, and fell all in a heap, and could not rise. When I arrived I found the animal lying upon his sternum in the usual manner, but with his muzzle pushed on to the ground, grinding his teeth, and in a comatose condition. Pulse and temperature fairly normal, the former 48 and the latter 101°. On looking around I noticed that he had evacuated a doughy mass covered with mucus.

I gave him : ℞.—Epsom salts, ℥xvi ; pot. bicarb., ʒiv ; pulv. zingib., ʒiv ; sprts. æth. nit., ℥ij ; tr. bellad., ℥j ; aqua, 2 quarts.

Ordered one-half tumbler of whiskey in two hours, and repeated in four hours. The animal has been hearty ever since.

